# Genome-Wide Analysis of Homeobox Gene Family in Legumes: Identification, Gene Duplication and Expression Profiling

**DOI:** 10.1371/journal.pone.0119198

**Published:** 2015-03-06

**Authors:** Annapurna Bhattacharjee, Rajesh Ghangal, Rohini Garg, Mukesh Jain

**Affiliations:** Functional and Applied Genomics Laboratory, National Institute of Plant Genome Research (NIPGR), Aruna Asaf Ali Marg, New Delhi, India; RIKEN Center for Sustainable Resource Science, JAPAN

## Abstract

Homeobox genes encode transcription factors that are known to play a major role in different aspects of plant growth and development. In the present study, we identified homeobox genes belonging to 14 different classes in five legume species, including chickpea, soybean, *Medicago*, *Lotus* and pigeonpea. The characteristic differences within homeodomain sequences among various classes of homeobox gene family were quite evident. Genome-wide expression analysis using publicly available datasets (RNA-seq and microarray) indicated that homeobox genes are differentially expressed in various tissues/developmental stages and under stress conditions in different legumes. We validated the differential expression of selected chickpea homeobox genes via quantitative reverse transcription polymerase chain reaction. Genome duplication analysis in soybean indicated that segmental duplication has significantly contributed in the expansion of homeobox gene family. The Ka/Ks ratio of duplicated homeobox genes in soybean showed that several members of this family have undergone purifying selection. Moreover, expression profiling indicated that duplicated genes might have been retained due to sub-functionalization. The genome-wide identification and comprehensive gene expression profiling of homeobox gene family members in legumes will provide opportunities for functional analysis to unravel their exact role in plant growth and development.

## Introduction

Homeobox genes are known to play an important role in body plan specification of higher organisms during early stages of embryogenesis. Initially, homeobox genes were isolated from the fruit fly, *Drosophila melanogaster*, but later these genes were identified in diverse organisms, like nematodes, fungi, plants and humans [1,2]. Homeobox genes encode a conserved 60 amino acid (aa) long DNA-binding domain, known as homeodomain (HD). In animal and plant genomes, homeobox genes are represented by a large gene family [2,3]. The characteristic three-dimensional structure of HD contains three alpha-helices, of which the second and third helices form a helix-turn-helix DNA-binding motif [4,5].

Based on the conserved amino acid sequence of HD along with the presence of other characteristic motifs, homeobox genes have been categorized into different groups. There are “typical” HD, characterized by a length of 60 amino acids and “atypical” HD having variation in amino acid length [6]. One such “atypical” HD has been named TALE (Three Amino acid Loop Extension) which is of 63 aa, having three extra residues between helices 1 and 2 [7,8]. Earlier, HD proteins were classified into the following classes; namely, KNOX, BELL, ZM-HOX, HAT, AT-HB8 and GL2 [9]. In another study, homeobox genes in rice were classified into ten subfamilies, namely HD-Zip I, HD-Zip II, HD-Zip III, HD-Zip IV, BLH, KNOX I, KNOX II, WOX, ZF-HD and PHD [10]. Furthermore, a comprehensive study on plant homeobox genes was also conducted where they were classified into 14 classes, including some new classes, such as NDX, DDT, PHD, LD, SAWADEE and PINTOX [3].

Members of plant homeobox gene family are known to participate in several developmental processes. Many members of HD-Zip I class are critical components regulating cotyledon development, leaf cell fate determination and blue light signalling [11,12]. Some HD-Zip II class members are involved in shade avoidance responses [13]. The members of HD-Zip III class are involved in apical meristem formation, vascular development and maintenance of adaxial or abaxial polarity of leaves and embryo [14]. HD-Zip IV proteins play primary role in the formation of outer cell layers of plant organs, in addition to controlling processes of anthocyanin pigmentation and maintenance of epidermal layer [15,16]. KNOX family members are known to have well-defined roles in shoot apical meristem maintenance [17]. They have been reported to interact with BEL family members to regulate hormone homeostasis [18]. WOX family members in *Arabidopsis* mark cell fate during early embryonic patterning and some of the members are known to be involved in stem cell maintenance and organogenesis [19,20]. WUSCHEL protein has been linked with cell differentiation during anther development [21]. The ZF-HD family members have been implicated in floral developmental processes in *Arabidopsis* [22]. Interestingly, a member of NDX class in soybean showed cell-specific expression pattern in nodules, highlighting its role in nodule development [23].

Legumes are important crop plants possessing the unique ability to fix atmospheric nitrogen. In addition, being rich source of proteins, legumes are very important for human diet. Although, homeobox genes have been identified in various plant species and characterized to some extent [3,10,24–26], their genome level analysis in legumes is lacking as of now. Recently, genome sequences of many legume plants have become available, which provided an opportunity for detailed characterization of homeobox genes in this important family of plants. In the present study, we identified homeobox genes in five legumes, including chickpea (*Cicer arietinum*), soybean (*Glycine max*), *Medicago* (*Medicago truncatula*), *Lotus* (*Lotus japonicus*) and pigeonpea (*Cajanus cajan*). On the basis of domain architecture and phylogenetic relationship, homeobox genes identified in legumes were classified into 14 different classes. Expression profiles of these genes were examined in different tissues/organs during various stages of development and in response to environmental cues. Moreover, analysis of whole genome duplication events provided insights into the expansion of soybean homeobox gene family. This study furnishes valuable information about homeobox gene family in legumes to facilitate functional analysis.

## Materials and Methods

### Screening of genomic resources for identification of homeobox genes in legumes

Homeobox genes in rice and *Arabidopsis* were retrieved from the previous studies [3, 10], and a non-redundant set of homeobox genes were retained for analysis (S1 Table). Proteome sequences of legume crops, chickpea (CGAP_v1.0: http://nipgr.res.in/CGAP/home.php) [27], soybean (Gmax_189; ftp://ftp.jgi-psf.org/pub/compgen/phytozome/v9.0/Gmax) [28], *Medicago* (Mtruncatula_198; ftp://ftp.jgi-psf.org/pub/compgen/phytozome/v9.0/Mtruncatula) [29], *Lotus* (build 2.5; www.kazusa.or.jp/*Lotus*/) [30] and pigeonpea (v-5.0; www.icrisat.org/gt-bt/iipg/home.html) [31] were downloaded from their respective databases. The homeobox protein sequences from *Arabidopsis* and rice were taken as query and searched against proteomes of different legumes via BLASTP. In addition, proteomes of legumes were searched against hidden Markov model (HMM) profiles of homeobox domain (PF00046) and zinc-finger HD (PF04770) via HMMER. Both similarity searches were performed at an e-value cut-off of ± 1e-05. The protein sequences obtained from above two approaches were concatenated and redundant entries were removed in order to create a non-redundant set of putative homeobox proteins for each legume. To confirm the presence of HD and identify other conserved domains, homeobox proteins from each legume were further subjected to domain search via SMART (www.smart.embl-heidelberg.de) and Pfam (www.pfam.sanger.ac.uk).

### Phylogenetic analysis and identification of conserved motifs

Multiple sequence alignment tool, CLUSTALX (v2.1; www.clustal.org/clustal2), was employed and phylogenetic trees were constructed using the neighbour-joining (NJ) method [32]. A gap open penalty of 10 and gap extension penalty of 0.2 were used for sequence alignment. Bootstrap analysis was performed using 1000 replicates and the tree was visualized using FigTree (v1.4.0; www.tree.bio.ed.ac.uk/software/figtree/). Conserved motifs other than HD, present in different classes of HD proteins were identified using MEME (Multiple EM for Motif Elicitation) Suite and viewed by MAST (Motif Alignment and Search Tool).

### Expression profiling of homeobox genes in legumes

We analyzed the expression patterns of homeobox genes from RNA-seq experiments conducted previously in soybean [33,34] and chickpea [27,35]. To study the expression profiles of soybean homeobox genes in response to biotic and abiotic stresses, the microarray data, available in Genevestigator v.3 (https://www.genevestigator.com/gv/plant.jsp) and Gene Expression Omnibus (accession number GSE40627) were used. The expression patterns of homeobox genes in *Lotus* and *Medicago* were analyzed using *Lotus japonicus* gene expression atlas (LjGEA) [36] and *Medicago truncatula* gene expression atlas (MtGEA) [37], respectively. Probe set IDs corresponding to *Lotus* homeobox genes were identified by BLASTN utility available at LjGEA, whereas probe set IDs corresponding to *Medicago* homeobox genes were identified using online Plexdb Blast (BLASTN) utility (http://www.plexdb.org/). For genes with more than one probe set ID, the probe showing better e-value and higher identity was considered (S1 Table). For *Medicago*, we analyzed RNA-seq data of vegetative and reproductive tissues also from a previous study [29]. Normalized data obtained from different studies were log_2_ transformed to generate heat-maps using MultiExperiment Viewer (MeV) software (v4.8.1).

For quantitative reverse transcription polymerase chain reaction (qRT-PCR) analysis, chickpea (*Cicer arietinum* L. genotype ICC4958) seeds were grown as described previously [38]. Different chickpea tissues/organs (shoot, root, stem, mature leaf, mature flower and young pod) were collected from plants as described [27]. For desiccation and salinity stress treatments, 10-day-old chickpea seedlings were transferred on folds of tissue paper and beaker containing 150 mM NaCl solution, respectively, at 22±1°C. For cold treatment, the seedlings were kept in water at 4±1°C. The control seedlings were kept at 22±1°C as described [35]. Root and shoot tissues were collected from stressed and control seedlings after 5 h of treatment. At least two independent biological replicates of each tissue sample were harvested and total RNA was isolated using TRI reagent (Sigma Life Sciences) according to the manufacturer’s instructions. Assessment of the quality and quantity of each RNA sample was done using NanoVue (GE Healthcare). Sequences of primer pairs used in this study have been listed in S2 Table. The qRT-PCR was performed following the protocol described previously [38]. The transcript level of each gene in different tissue samples was normalized with the transcript level of the most suitable internal control gene, elongation factor 1-alpha (*EF-1a*) [38]. The correlation between expression profiles of selected genes obtained from qRT-PCR and RNA-seq analysis was determined using R programming environment.

### Identification of *cis*-regulatory elements in chickpea and soybean

Genomic coordinates of chickpea and soybean genes were determined from genome annotation file (gff file) and the promoter sequence (2 kb) of each gene was retrieved using in-house perl script from their respective genome sequences. *Cis*-regulatory elements present in the promoter sequence of homeobox genes were scanned at PLACE web server (http://www.dna.affrc.go.jp/). In addition, the known binding sites/motifs of HD-Zip I (AH1, CAAT(A/T)ATTG and/or AH2, CAAT(C/G)ATTG) and of HD-Zip II (AH2) class homeobox proteins were identified in the promoter sequences of all chickpea and soybean genes using custom perl script. Further, coexpression analysis of HD-Zip I and HD-Zip II subfamily genes in chickpea and soybean with genes harbouring AH1 and/or AH2 motifs in their promoter regions was carried out using R programming [33–35]. The genes with a Pearson correlation coefficient ≥ 0.7 and p-value of ≤ 0.05 were designated as significantly correlated.

### Genome localization and gene duplication

To determine the location of homeobox genes onto chromosomes, coordinates of individual genes were obtained from genome annotation file (gff file) of respective legumes. The list of homeobox genes in duplicated genomic regions and Ka/Ks values for each duplicated gene for soybean were retrieved from batch download option of Plant Genome Duplication Database (PGDD; http://chibba.agtec.uga.edu/). The Ks values have been calculated using Nei-Gojobori method implemented in PAML package following CLUSTALW and PAL2NAL alignments. The duplicated homeobox genes in soybean were visualized using Circos software (http://circos.ca/). The expression patterns of homeologous homeobox genes were extracted from RNA-seq data as described earlier.

## Results and Discussion

### Homeobox genes in legumes and their classification

**Fig 1:**
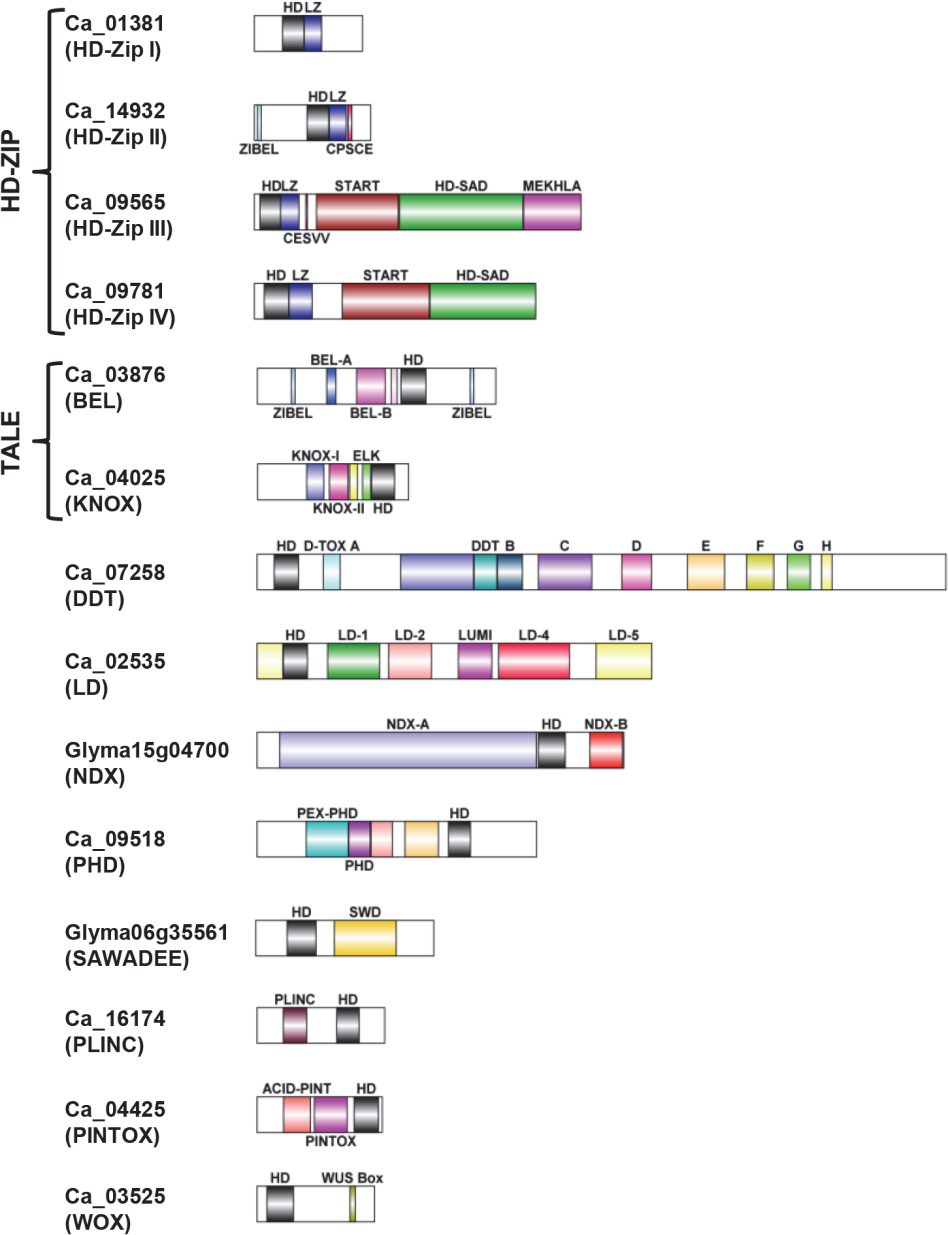
Diagrammatic representation of the domain architecture of all the 14 classes identified in legume homeobox proteins. Each class is represented with an example of a chickpea/soybean homeobox protein. Different domains and motifs have been indicated with different colors, homeobox domain (HD), leucine-Zipper (LZ), ZIBEL motif, CPSCE motif, CESVV motif, START domain, HD-START associated domain (HD-SAD), MEKHLA domain, DDT domain, LUMI, conserved motifs in LD HD proteins (LD1, LD2, LD4 and LD5), NDX domain (A and B), PEX-PHD, PHD, BEL domain (A and B), SAWADEE (SWD), KNOX domain (I and II), ELK motif, zinc-finger (PLINC) and WUS box (WOX). D-TOX A is indicated with its full symbol; D-TOX B, D-TOX C, D-TOX D, D-TOX E, D-TOX F, D-TOX G and D-TOX H are indicated as B, C, D, E, F, G and H, respectively.

The non-redundant set of homeobox genes from *Arabidopsis* (113) and rice (113) belonging to 14 classes were extracted from previous reports (S1 Table). Based on BLASTP and HMM profile searches followed by confirmation of the presence of HD, we identified a total of 89 homeobox genes in chickpea, 276 in soybean, 82 in *Medicago*, 92 in *Lotus* and 137 in pigeonpea (Table 1). The homeobox genes in legumes were found to be distributed in 14 different classes, including two superclasses, i.e. HD-Zip (HD-Zip I, HD-Zip II, HD-Zip III and HD-Zip IV) and TALE (KNOX and BEL), and eight classes, i.e. PLINC (ZF-HD), WOX, DDT, PHD, NDX, LD, PINTOX and SAWADEE as reported previously for plants [3]. A schematic representation of domain composition of different classes of homeobox genes is depicted in Fig. 1. The total number of homeobox genes identified in soybean was the highest (276 genes). At least one member was identified for each of the 14 classes in soybean, *Medicago* and pigeonpea, whereas homeobox genes of chickpea were distributed in 12 classes (no member in NDX and SAWADEE classes). In *Lotus*, there was no member identified for LD and NDX classes, and one member was kept under the category of “unclassified”, as it did not possess any known characteristic domain other than HD (Table 1). Identification of lesser number of homeobox genes in chickpea (89) and *Lotus* (93), may be due to their incomplete (~70%) draft genome sequence available as of now.

**Table 1:** Classification of homeobox gene family members in different legumes, *Arabidopsis* and rice.

Class	Chickpea	Soybean	*Medicago*	*Lotus*	Pigeonpea	*Arabidopsis*	Rice
**HD-ZIP I**	12	35	11	9	18	17	14
**HD-ZIP II**	9	27	7	9	14	10	14
**HD-ZIP III**	5	12	5	4	6	5	9
**HD-ZIP IV**	8	31	15	16	15	16	12
**PLINC**	14	51	14	23	20	17	14
**WOX**	14	33	10	12	19	16	14
**BEL**	12	34	4	6	17	13	14
**KNOX**	5	28	6	5	16	8	12
**DDT**	6	13	5	3	5	4	3
**PHD**	2	6	1	2	2	2	2
**PINTOX**	1	2	1	1	1	1	1
**LD**	1	1	1	0	1	1	1
**NDX**	0	1	2	0	1	1	1
**SAWADEE**	0	2	1	2	2	2	2
**Unclassified**	0	0	0	1	0	0	0
**Total**	**89**	**276**	**83**	**93**	**137**	**113**	**113**

Number of members identified in each class (based on domain composition and phylogenetic relationship) are given for each plant.

The members of homeobox gene family have been predicted in different legumes and are available in databases, namely PlantTFDB and LegumeTFDB. However, the number of homeobox genes reported in our study is much higher. PlantTFDB reports the existence of 77 homeobox genes in chickpea, 183 in soybean, 62 in *Medicago*, 64 in *Lotus* and 82 in pigeonpea, whereas LegumeTFDB shows the presence of 260 homeobox genes in soybean, 62 in *Medicago* and 80 in *Lotus*. Moreover, SoybeanTFDB reports the existence of 269 homeobox proteins in soybean as compared to 276 homeobox proteins identified in our study. Overall, these differences may be due to the robust methodology of identification employed or latest versions of the genome annotation used in our study. The complete details, including gene identifier, classification, conserved domain(s), genomic location, protein length and genomic coordinates of the homeobox genes identified in different legumes are enlisted in S1 Table.

Super-class HD-Zip was found to have the maximum representative members among homeobox genes in legumes similar to other plants. We identified 105 members of HD-Zip superclass in soybean as compared to 88 members in a previous report [39]. This difference in number of HD-Zip proteins may be attributed to more robust methodology employed for identification in our study. In HD-Zip superclass, leucine-Zipper (LZ) domain is known to mediate protein-protein interactions. Additional characteristic domains present besides HD are known to perform specific functions as well. CPSCE motif in HD-Zip II class acts as a redox sensor [40], ZIBEL motif mediates interaction between HD-Zip II proteins and BEL HD proteins or similar targets [3]. In HD-Zip III class, MEKHLA domain is speculated to be involved in oxygen redox and light signalling [41]. HD-Zip IV proteins containing START (STeroidogenic Acute Regulatory protein-related lipid Transfer) domain and HD-SAD (START Associated conserved Domain) domain (Fig. 1) possess putative lipid binding capability [42] and transcriptional activation property, respectively [43].

**Fig 2:**
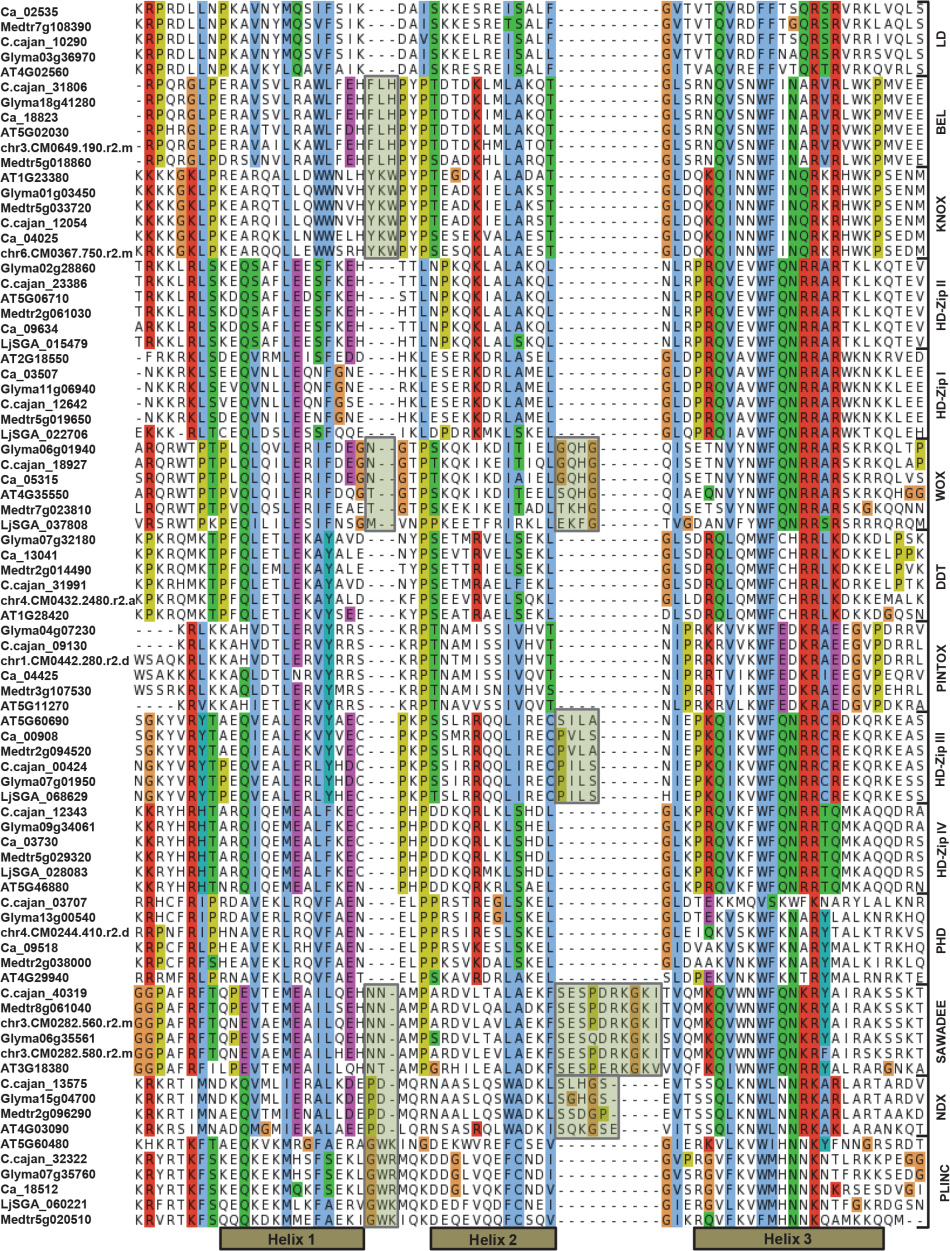
Multiple sequence alignment of amino acid (aa) sequences of HD from different classes. The representatives of each class from *Arabidopsis thaliana* (AT), *Cicer arientinum* (Ca), *Glycine max* (Glyma), *Cajanus cajan* (C. cajan), *Medicago truncatula* (Medtr) and *Lotus japonicus* (LjSGA, chr) have been shown. The alignment was obtained using CLUSTALX and conserved amino acids of different physicochemical properties are highlighted in different shades using the Jalview software. Atypical aa residues are also shaded and the positions of three alpha helices are indicated at the bottom of the diagram.

The second largest class of homeobox proteins was represented by PLINC (Plant Zinc Finger, previously called ZF-HD). These proteins contain two highly conserved zinc-finger-like motifs upstream to HD (Fig. 1), which are involved in protein-protein interaction by mediating homo- and hetero-dimerization [44]. Maximum members of PLINC class are present in soybean (51) followed by *Lotus* (23) and pigeonpea (20) (Table 1). KNOX and BEL class HD proteins belonging to the superclass TALE harbor three extra amino acid residues (just before PYP) in the loop connecting the first and second helices of the HD (Fig. 2). In total, 73 and 60 proteins of legumes were classified into BEL and KNOX classes, respectively (Table 1). BEL or BEL1-like homeobox (BLH) class proteins harbor a domain of unknown function, called POX domain (S1 Table) towards N-terminal of HD. It has been proposed that this co-domain is a bipartite domain composed of BEL-A and BEL-B (Fig. 1) [3]. We detected a highly conserved 10 aa motif named “ZIBEL” present at both the ends (C-terminal and N-terminal) of BEL proteins (Fig. 1). KNOX domain residing towards N-terminal, is composed of two conserved stretches (KNOX A/I and KNOX B/II, separated by a variable region) and ELK domain upstream to HD (Fig. 1) [7]. The ELK, KNOX A and KNOX B domains are required for nuclear localization, target gene suppression and homo-dimerization, respectively [45,46]. Notably, the number of TALE superclass proteins was significantly lesser (10) in *Medicago* as compared to other legumes (Table 1). The biological significance of this difference in *Medicago* remains to be elucidated.

WOX proteins contain one extra residue between helices 1 and 2, and four extra residues between helices 2 and 3 (Fig. 2). WUS-box motif, a sequence of eight conserved residues (TLPLFPMH) is present towards C-terminus of HD (Fig. 1) [20]. WOX proteins showed presence of an acidic amino acid stretch between HD and WUS box apart from other distinctive conserved motifs. DDT homeobox proteins harbor eight additional conserved motifs, named D-TOX A to H, distributed over the entire length of the protein, in addition to the DDT domain (Fig. 1). This class consists of longest plant homeobox proteins with sequences of length up to ~1800 aa (S1 Table). In plants, classification of DDT proteins was done into three subclasses (D-TOX 1, D-TOX 2 and D-TOX 3) [3]. Among these, D-TOX 3 was considered as eudicot-specific that has lost all characteristic motifs of DDT class except D-TOX A motif. We also made similar observations within DDT class, where one of the two major clades of DDT (adjacent to BEL in S1 Fig.) had members harboring only D-TOX A motif. The maximum length of this group of DDT proteins was of 546 aa, thereby deviating from basic characteristics of DDT HD class.

### Chromosomal localization and phylogenetic analysis

Chromosomal localization of all the homeobox genes was analyzed in different legumes (S1 Table). It was seen that *Arabidopsis* and rice homeobox genes were unevenly distributed on the chromosomes (S1 Table). In legumes, apart from soybean and *Medicago*, a large number of homeobox genes in chickpea, *Lotus* and pigeonpea were found to be mapped to unanchored scaffolds. Out of 89 chickpea homeobox genes, less than 45% (38 genes) were assigned to eight chickpea linkage groups, while the rest were located on scaffolds. This percentage was higher for pigeonpea, where 82 of the 137 homeobox genes mapped onto eleven linkage groups (S1 Table). This may be due to availability of incomplete genome sequence of these legumes. All but two soybean homeobox genes were located on the twenty chromosomes. On the other hand, 82 of 83 homeobox genes of *Medicago* were found distributed on eight chromosomes, with chromosome 6 harboring only two genes (S1 Table). Evidently, some chromosomes in these legumes show sparsely situated homeobox genes, whereas some chromosomes possess dense distribution of homeobox genes.

**Fig 3:**
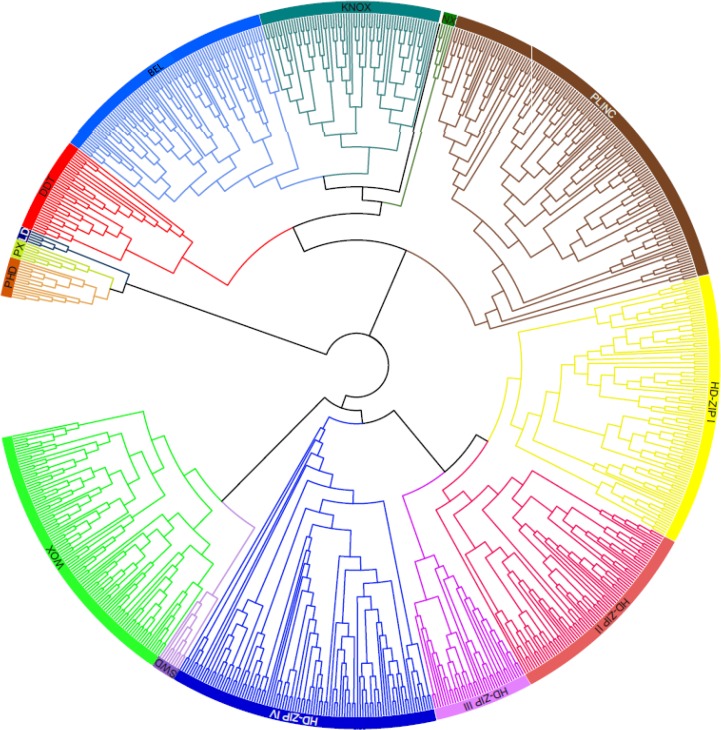
Phylogenetic tree based on full-length homeobox protein sequences identified in *Arabidopsis*, chickpea, soybean, *Medicago*, *Lotus* and pigeonpea. The phylogenetic tree is unrooted and bootstrap support is based on 1000 replicates. Classes of homeobox gene family (labeled) are well separated in different clades in this analysis and are consistently supported by conserved, class-specific domain architecture.

To study the evolutionary relationship among homeobox proteins, an unrooted phylogenetic tree was constructed after multiple sequence alignment of full-length 791 homeobox proteins from five legumes and *Arabidopsis* using CLUSTALX. The members of homeobox gene family were distinctly clustered into 14 classes (Fig. 3), supporting our domain composition based classification. The detailed phylogenetic tree with bootstrap values and gene identifiers has been presented in S1 Fig. The phylogenetic tree generated using only the homeobox domain sequences also supported the clustering of almost all the proteins in 14 classes (S2 Fig.). Phylogenetic relationship also revealed that many homeobox proteins representing various classes showed very high homology within or across the species (S1 and S2 Figs.). It has been suggested that proteins with higher homology within a class/subfamily may perform similar functions [47].

### Differential expression of homeobox genes during development

**Fig 4:**
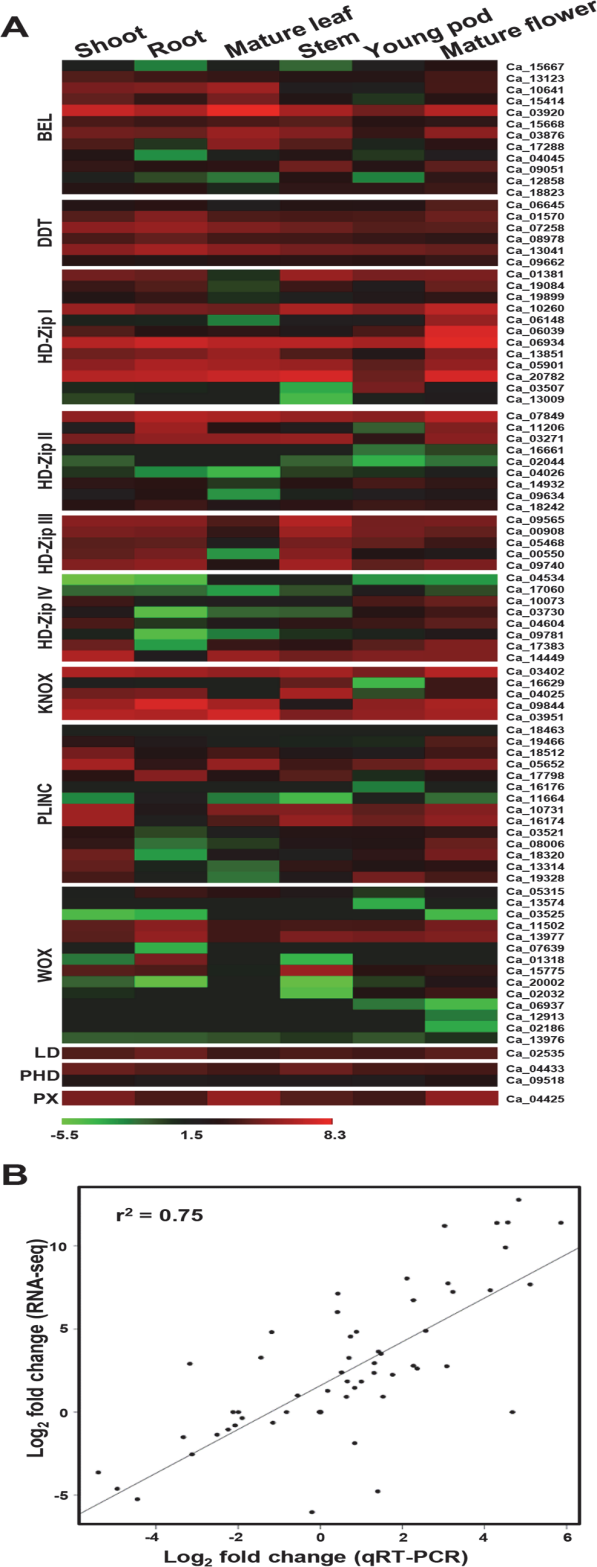
Differential gene expression of chickpea homeobox genes in various tissues/organs. (A) Heat-map showing expression patterns of homeobox genes in different tissues/organs. The scale at the bottom represents log_2_ RPKM value. The maximum value is displayed as dark red and minimum value is displayed as light green. Gene IDs are given on right side. (B) The correlation between gene expression results obtained from RNA-seq and qRT-PCR analysis. Each data point represents log_2_ of RPKM value for RNA-seq and qRT-PCR.

To gain insights into the putative function of homeobox genes in different legumes, their expression patterns were analyzed in various tissues/organs/developmental stages. In chickpea, RNA-seq data [35] analysis revealed differential expression of homeobox genes in several tissues and/or organs. Homeobox genes of four classes, namely DDT, LD, PHD and PINTOX showed more or less uniform expression pattern across all the tissues/organs analyzed (Fig. 4A). The members of HD-Zip I class were found to be highly up-regulated in mature flower, thereby suggesting their role in flower development. Previously, it has been reported that *Arabidopsis* HD-Zip I members are expressed in diverse developmental stages, with only few genes showing tissue/organ specific expression. For example, *ATHB53* was specifically expressed in roots and flowers and *ATHB13* was detected in seedling, leaves and flowers only [11]. Many of the HD-Zip II, HD-Zip IV and WOX class genes were found to be expressed at lower level in the chickpea tissues/organs analyzed. Most prominently, expression patterns of HD-Zip IV genes showed considerable down-regulation in roots of chickpea (Fig. 4A). A few chickpea homeobox genes exhibited tissue-specific/preferential gene expression as well. For example, Ca_17060 (HD-Zip IV member) was expressed in young pod, Ca_02032 and Ca01318 (WOX family members) were expressed in young pod and flower, and root, respectively. HD-Zip IV members are known to be involved in shoot and reproductive developmental processes besides maintenance of epidermal cell layer [15]. GLABRA2 (GL2), a HD-Zip IV member in *Arabidopsis*, was found to play a crucial role in root hair development in addition to leaf epidermis patterning [48]. Interestingly, many WOX class members have also been implicated in root and flower development in *Arabidopsis* [49]. To validate the results of differential gene expression analysis obtained from RNA-seq data, we performed qRT-PCR analysis of at least 11 randomly selected differentially expressed genes in six different tissues/organs of chickpea. qRT-PCR analysis revealed similar expression patterns of all the selected genes as observed in RNA-seq data. The statistical analysis also showed a very good agreement (correlation coefficient of 0.75) between the results of qRT-PCR and RNA-seq data analysis (Fig. 4B).

For soybean, we investigated the global expression profile of homeobox genes using the previously reported RNA-seq data [33,34]. The homeobox genes in soybean also exhibited diverse expression patterns, including low to high level, tissue-specific and/or preferential expression in one or more tissue sample analyzed. Except members of HD-Zip superclass (HD-Zip I-IV) and few members of PLINC and DDT class, homeobox proteins were found to be expressed at low levels in root tip (S3 Fig.). Nearly all the HD-Zip IV proteins, except Glyma08g09430, Glyma15g13950, Glyma09g02990, Glyma09g03000 and Glyma08g09440, were up-regulated in shoot apical meristem, thereby suggesting their role in shoot apical meristem maintenance. In *Arabidopsis*, HD-Zip III members were found to be responsible for shoot apical meristem maintenance and polarisation of leaf cell primordia [14,50]. Only PLINC and few HD-Zip I members were found to be active in different stages of seed and pod shell development in soybean. It was previously also reported that members of PLINC class coordinate floral development in *Arabidopsis* [22]. In addition, a fraction of soybean BEL proteins were expressed at moderate levels in later stages of seed development (S3 Fig.). However, BEL proteins were highly expressed in nodule, flower and leaf. In fact, BEL family members, along with HD-Zip-I, KNOX and DDT genes were found to be up-regulated in nodules, thereby suggesting their active participation in root nodule development related biological processes. WOX proteins were least expressed among soybean homeobox gene family members in different tissue/organ/developmental stages (S3 Fig.). Interestingly, it has recently been reported that a BEL1-type homeobox gene, *SH5*, induces seed shattering by development of abscission zone and suppression of lignin biosynthesis [51].

For expression profiling of *Medicago* and *Lotus* homeobox proteins, microarray data from their respective gene expression atlases were analyzed. In *Lotus*, expression analysis of different tissue/organ and developmental stages of pods and seeds were undertaken. Interestingly, one member of PLINC class of *Lotus*, chr2.CM1835.100.r2.m, was highly up-regulated in developmental stages of pod and seed (S4A Fig.). Similarly, few members of PLINC class in *Medicago* were also found to be up-regulated in developmental stages of pods and seeds (S4B Fig.). These observations suggest that PLINC proteins may have a conclusive role pertaining to seed setting in legume pods. A recent study in *Medicago truncatula* highlighted the role of several members of HD-Zip class, WOX and KNOX class in early embryo development [52].

### Genome duplication and expression patterns of duplicated homeobox genes

**Fig 5:**
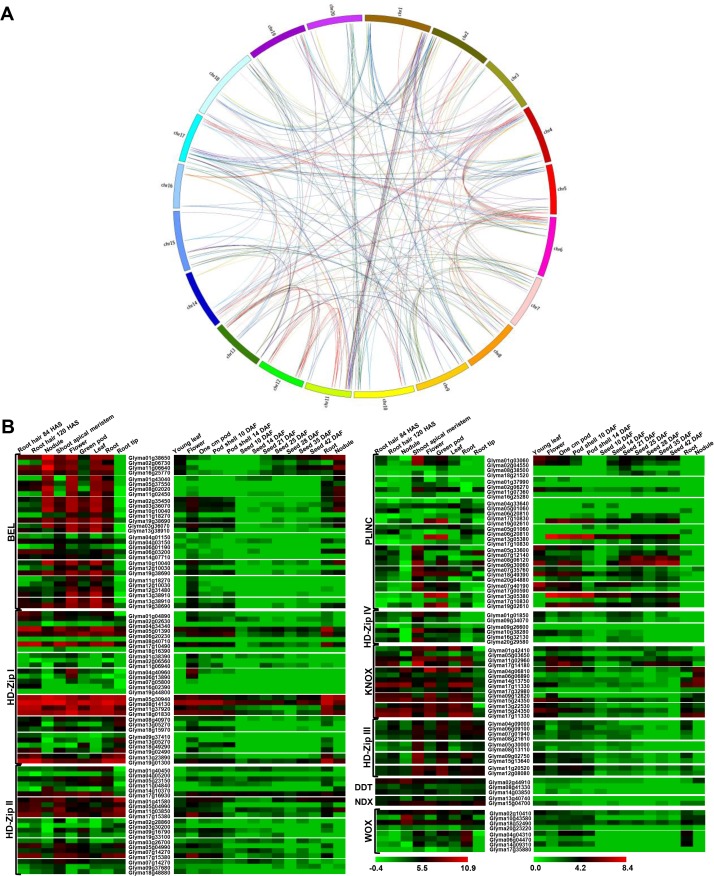
Gene duplication events in homeobox gene family in soybean. (A) Circos diagram showing the genic position of 328 gene pairs on soybean chromosomes. Homeobox gene pairs present on duplicated chromosomal segments are connected by different colored lines according to different classes (B) Heat-map showing remarkable differential expression patterns among duplicated gene pairs in different tissues/organs/developmental stages. Genes have been grouped on the basis of class to which they belong. The scale at the bottom represents log_2_ RPKM value. The maximum value is displayed as dark red and minimum value is displayed as light green.

Whole genome duplication events in plants have been considered as a mechanism of diversification and adaptation to the environment [53]. However, functions of duplicated genes are poorly understood. In legumes, soybean has undergone genome duplication two times (59 and 13 million years ago), which resulted in emergence of multiple copies of ~75% of soybean genes [28]. Relatively higher number of homeobox genes identified in soybean as compared to other legume species may be due to these whole genome duplication events. We found that members of homeobox gene family are distributed preferentially in duplicated blocks in soybean (S4 Table). A total of 246 (89.1%) homeobox genes in soybean were found located on duplicated chromosomal blocks. Interestingly, we could not locate even a single event of tandem duplication in soybean. These observations suggest that segmental duplication has played an important role in expansion of soybean homeobox gene family, since this process allows retention of numerous duplicated genes in the genome [54]. The duplicated gene pairs of respective classes of homeobox gene family have been represented pictorially in Fig. 5A. The non-synonymous/synonymous substitution ratio (Ka/Ks) tells us about the selective evolutionary pressure acting on a gene. Majority (96.3%) of the gene pairs were found to have Ka/Ks < 1 suggesting their evolution to be under the influence of purifying selection (S4 Table). The purifying selection has also been previously observed for HD-Zip proteins in soybean [39] and poplar [26].

Events of gene duplication may serve as a crucial mechanism to increase the functional diversity of gene family due to spatial and/or temporal changes in gene expression. Differences in gene expression pattern may result from non-functionalization, sub-functionalization or neo-functionalization of duplicated genes. Similar evidences have been reported in other model plants like *Arabidopsis* [55]. We observed that majority of soybean homeobox family duplicated genes were differentially expressed in tissue/organ/developmental stages analyzed (S5 Fig.). Likewise, differential expression of nearly 50% of 17547 duplicated genes in soybean was observed across seven tissues, thereby suggesting sub-functionalization [56]. Based on gene expression patterns, we observed three types of functional variations in homeologous gene pairs in soybean. For instance, sub-functionalization was observed in Glyma01g38650/Glyma02g06730 with no expression of Glyma02g06730 in root hairs. In Glyma10g10040/Glyma12g10030 gene pair, Glyma12g10030 expression decreased to basal level in root, root hair and nodule in contrast to Glyma10g10040. In addition, instances of neo-functionalization were observed in Glyma04g01150/Glyma04g03150 and Glyma04g01150/Glyma06g03200 gene pairs, where expression of Glyma04g03150 and Glyma06g03200 could be detected in root hairs and nodules, contrary to Glyma04g01150. The phenomenon of non-functionalization was exhibited by Glyma04g33640/Glyma19g02610 gene pair, where Glyma19g02610 was expressed in reproductive tissues, however, Glyma04g33640, did not show expression in most of the developmental stages analyzed (Fig. 5B). These observations imply that the evolutionary fate of soybean homeobox genes have been closely regulated by gene duplication events. Overall, these analyses indicate that purifying selection has majorly contributed in retention and maintenance of duplicated gene pairs during evolution. Moreover, expression profiling of duplicated soybean homeobox proteins highlighted that majority of them have undergone sub-functionalization. Such observations are consistent with other plant species, where closely related genes, have been shown to display diverse expression patterns [10,39].

### Differential expression of homeobox genes in response to abiotic and biotic stresses

**Fig 6:**
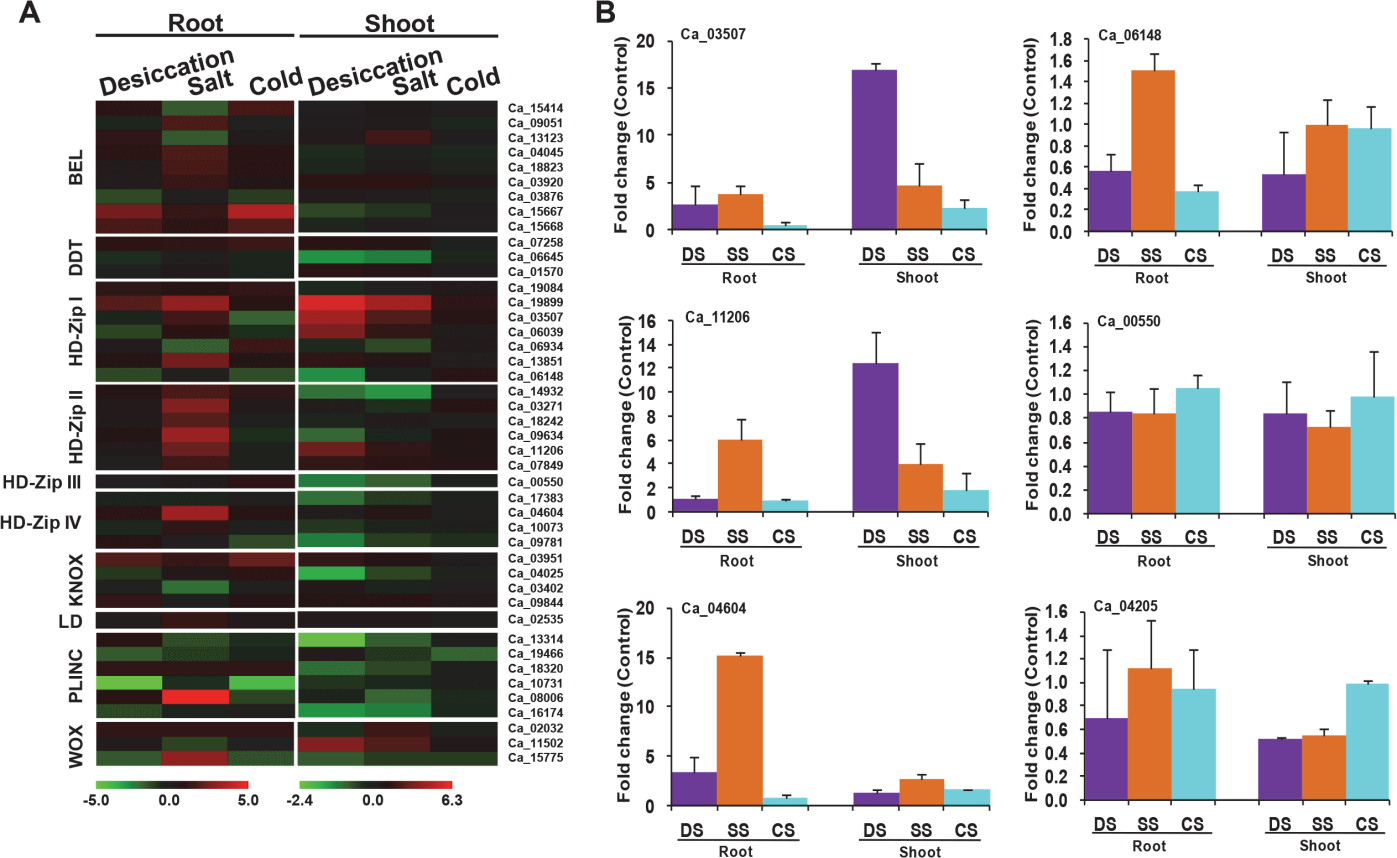
Differential expression of chickpea homeobox genes under abiotic stress conditions. (A) Heat-map showing differential expression of chickpea homeobox genes under abiotic stress conditions in root and shoot tissues. The scale at the bottom represents log_2_ fold change, maximum value is displayed as dark red and minimum value is displayed as light green. Gene IDs are given on right side. (B) Real-time PCR analysis to validate the differential expression of representative chickpea homeobox genes during various abiotic stress conditions. The mRNA levels for each candidate gene were calculated relative to its expression in control root or shoot tissues. DS, desiccation stress; SS, salinity stress; CS, cold stress.

Crop production is often adversely affected by several abiotic stress factors like desiccation, salinity and extremes of temperatures. Since homeobox genes are known to play an important role in abiotic stress responses, we analyzed the expression profile of chickpea homeobox genes in root and shoot tissues subjected to desiccation, salinity and cold stresses using RNA-seq data [35]. Out of 89 chickpea homeobox genes, 44 were found to be significantly differentially regulated in root and/or shoot tissues subjected to at least one of the abiotic stress conditions. Overall, more number of homeobox genes were up-regulated in root tissues subjected to salinity stress as compared to desiccation and cold stresses. All HD-Zip II members were reasonably up-regulated under salinity stress in root tissues, thereby suggesting their possible role in salinity stress responses (Fig. 6A). However, HD-Zip II genes showed no change in their expression pattern in root tissues under desiccation stress. Highest fold change in root tissue was recorded for Ca_08006, a member of PLINC class, when subjected to salinity stress. Cold stress did not alter the expression level of most homeobox genes. Only a few members showed differential expression in root tissues in response to cold. However, no considerable alteration in transcript levels could be detected in shoot tissues under cold stress. Under desiccation stress, HD-Zip I genes were highly up-regulated in shoot tissues as compared to root tissues (Fig. 6A). Similar observations have been made in *Arabidopsis*, where transcript levels of HD-Zip I members (*ATHB7* and *ATHB12*) increased tremendously in response to desiccation stress [57]. However, Ca_06148 and Ca_00550 were found to be downregulated in response to all the abiotic stress conditions analyzed in either of the tissues. Highest upregulation in shoot tissue was recorded for Ca_19899, a member of HD-Zip I class, when subjected to desiccation stress. However, this gene was greatly up-regulated during salinity stress as compared to desiccation stress in root tissues (Fig. 6A). It has been reported that a cotton homeobox gene, *GhHB1*, is specifically expressed in root tissues and gets up-regulated under exogenous salinity treatment [58]. Similarly, differential expression of many homeobox genes during abiotic stress conditions has been reported in various plant species [10,24,35,59]. We performed qRT-PCR analysis of six randomly selected differentially expressed homeobox genes in root and shoot tissues of chickpea during desiccation, salinity and cold stress conditions to validate the results obtained from RNA-seq data (Fig. 6B). The qRT-PCR analysis revealed similar differential expression patterns of all the selected genes as observed in RNA-seq data showing good correlation between the results of qRT-PCR and RNA-seq data analysis. These results suggest that homeobox genes may prove to be suitable candidates for engineering abiotic stress tolerance in crop plants.

**Fig 7:**
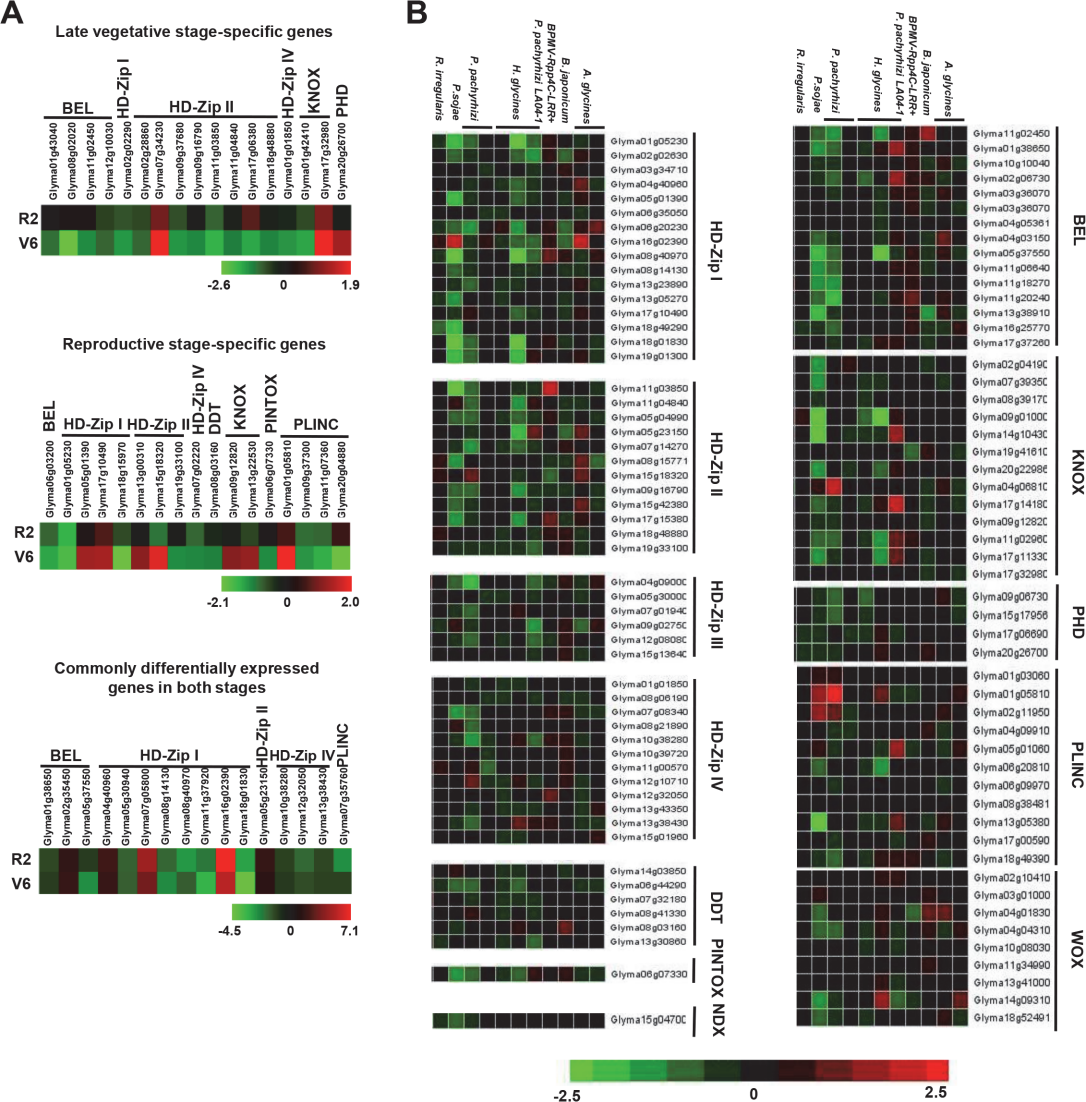
Differential expression of soybean homeobox genes under abiotic and biotic stress conditions. (A) Heat-map showing differential expression patterns of soybean homeobox genes during drought stress condition at late vegetative (V6), full bloom reproductive (R2) and both stages of development. The scale at the bottom represents log_2_ fold change value. The maximum value is displayed as dark red and minimum value is displayed as light green. Gene IDs are given on the right side. (B) Heat-map showing expression patterns of soybean homeobox genes under biotic stresses caused by various pathogens. The scale at the bottom represents log_2_ ratio of expression value. The maximum value is displayed as dark red and minimum value is displayed as light green. Images have been created and retrieved by Genevestigator v.3. Gene IDs are given on the top.

In a previous study, genome-wide transcriptome analysis reported the differential expression of several genes in soybean leaf tissue under drought stress at late developmental stages [60]. We utilised the microarray data from this study in order to understand the role of soybean homeobox genes in abiotic stress responses. Of the 276 soybean homeobox genes, 50 genes were found to be significantly differentially expressed in at least one of the conditions analysed. Among them, 17 genes were specifically differentially expressed at either late vegetative stage or full bloom reproductive stage and 16 homeobox genes were commonly differentially expressed at both the developmental stages of soybean (Fig. 7A).

Apart from abiotic stress factors, a wide range of biotic stress factors, like virus, bacteria, fungi, and nematode severely damage the crop productivity. Transcript levels of homeobox genes are altered under biotic stresses as well [61,62]. Hence, we analyzed the expression profile of soybean homeobox genes under biotic stress conditions using microarray data from Genevestigator v.3. Many soybean homeobox genes, were found to be significantly differentially regulated in response to at least one of the conditions analyzed. A maximum number of HD-Zip I class homeobox genes followed by BEL class and HD-Zip II class members were differentially expressed due to biotic stress factors (Fig. 7B). For example, among HD-Zip I proteins, Glyma16g02390 was found to be highly upregulated by *Aphis glycines* and *Phytopthora sojae* infection, whereas Glyma08g40970, Glyma18g01830, Glyma19g01300 and Glyma01g05230 were significantly downregulated in response to *Heterodera glycines* and *P*. *sojae* infection. Several KNOX members, namely, Glyma17g14180, Glyma04g06810, Glyma09g01000 and Glyma14gg37550 were differentially expressed in response to *H*. *glycines*, *Phakopsora pachyrhizi* and *P*. *sojae* infection. In addition, elevated transcript levels of some PLINC class members (Glyma01g03060, Glyma01g05810 and Glyma05g01060) in response to *P*. *pachyrhizi*, *P*. *sojae* and *H*. *glycines* infection, was also observed. The above expression profiling suggests a distinctive role of homeobox genes in biotic stress responses. As of now, only some reports have provided preliminary evidence of biotic stress-responsiveness of homeobox genes across various plant species and speculate their role in pathogen resistance [61,62]. Thus, the involvement of homeobox genes in pathogen-related responses needs to be explored in greater detail.

Transcription factors can act as master regulators as they can regulate the expression of several genes via binding to their promoter sequences. However, transcription factors may themselves be under the control of other upstream regulators, which may bind to promoter region of homeobox genes thereby regulating the cascade of reactions occurring during various biological processes in plants. We carried out a *cis*-regulatory element search in promoter regions (1 kb upstream) of homeobox genes in chickpea and soybean. Several *cis*-regulatory elements primarily known to be involved in various processes of plant development like leaf, shoot and root development were detected in the promoter sequences analysed (S3 Table). In addition, some seed specific *cis*-regulatory elements have been found in promoter regions of many homeobox genes. Further, existence of characteristic stress-responsive *cis*-regulatory elements, like ABRE, DRE and/or LTRE suggested stress-responsive regulation of these genes. Interestingly, auxin-responsive elements were also detected in the promoters of some homeobox genes in chickpea and soybean. The presence of such *cis*-regulatory elements suggests that homeobox genes may play pivotal role in various developmental processes, hormonal crosstalk and abiotic stress responses in legumes as well.

Overall, homeobox genes have been established as critical regulators of plant development. So far, many among them have emerged to play significant role in specific stress responses at various stages of plant development [10,25,57,62,63]. We also observed the differential/specific expression of many homeobox genes in different tissues/organ/developmental stage and abiotic/biotic stress conditions. Thus, homeobox genes are speculated to coordinate both developmental processes and stress-adaptive pathways in plants [10,64].

### Identification of putative downstream targets of HD-Zip I and HD-Zip II proteins

During abiotic stress conditions, some members of HD-Zip superclass are reported to bind specifically to *cis*-regulatory elements, thereby regulating the action of several downstream genes [65]. Investigations of DNA-binding specificities and dimerization properties of HD-Zip family members in *Arabidopsis* revealed that HD-Zip I members have the ability to bind to CAAT(A/T)ATTG (AH1) and/or CAAT(C/G)ATTG (AH2) motif(s), whereas HD-Zip II members can bind to AH2 motif [66,67].

Since, the binding sites of HD-Zip I and HD-Zip II class homeobox proteins are well documented, we scanned 2 kb upstream sequences of all the genes in soybean and chickpea to identify the presence of AH1 and/or AH2 motif(s). In total, 3,971 soybean genes were found to harbor at least one or more of these motifs, signifying the potential downstream targets of HD-Zip I and HD-Zip II class members. AH1 and/or AH2 motifs were present in promoters of 2671 and 1379 genes, respectively. A total of 1320 genes were found to harbor these motifs in chickpea. These genes are speculated to be probable target genes of HD-Zip I and HD-Zip II proteins. GOSlim analysis revealed that genes involved in various developmental processes, response to abiotic and biotic stress, and various enzymatic activities were most represented among the AH1 and/or AH2 motif harboring genes in their promoters (S6 Fig.), suggesting that these genes might be the putative targets of homeobox transcription factors. Coexpression analysis revealed 21 chickpea homeobox genes to be significantly (with Pearson correlation coefficient cut-off of 0.7 and p-value ≤ 0.05) coexpressed with 816 other chickpea genes harbouring AH1 and/or AH2 motifs, whereas, 152 soybean homeobox genes were found to be significantly coexpressed with 2093 soybean genes harbouring AH1 and/or AH2 motifs (S5 Table). Notably, in chickpea and soybean, 83 and 292 genes were found to be highly positively (≥ 0.95) correlated. At least, 113 chickpea genes showed significant negative correlation with homeobox genes (≤ -0.95) respectively, whereas not a single gene exhibited negative correlation ≤ (-0.90) with homeobox genes (S7 Fig.). The coexpression of a large number of other genes suggests the involvement of HD-Zip I and HD-Zip II class proteins in a complex transcriptional regulatory network responsible for various cellular processes. Due to lack of definite knowledge of binding specificity and suitable experimental evidence, such analysis could not be carried out for homeobox proteins belonging to other classes.

Interestingly, in rice, previous reports have established that HD-Zip I and HD-Zip II proteins bind to AH1 and/or AH2 motifs [68]. Very recently, it was also identified that an abiotic stress-responsive gene belonging to HD-Zip I class, *Oshox22*, could bind to either and/or AH2 motif(s) suggesting that homeobox genes across different plant species govern regulation of many downstream target genes via binding to AH1 and/or AH2 motif(s) [69]. In addition to abiotic stress responses, homeobox proteins possess ability to bind to *cis*-regulatory elements due to hormonal induction too. In *Medicago truncatula*, during lateral root emergence, a HD-Zip I family member, MtHB1, was shown to bind to AH1 motif located in promoter of LOB-like gene, *LBD1*, which is regulated transcriptionally by auxin [70]. The presence of such motifs in promoters of numerous genes suggests a preferential regulation via homeobox transcription factors. In future, these *in-silico* identifications of *cis*-regulatory elements would require experimental validation.

In conclusion, the comprehensive analysis of homeobox gene family members in legumes has generated a rich repertoire of knowledge for future investigation. Transcript profiling in legumes reiterated the diverse role of homeobox genes in biology of various tissues/organs/developmental stages and stress responses in legumes. Gene duplication analysis revealed that whole genome duplication events have resulted in expansion of homeobox gene family in soybean, which may have seemingly contributed to functional diversification in course of evolution. This fact was substantiated by analysis of expression profiles of duplicated soybean homeobox genes. Overall, the current study has built a foundation to initiate detailed investigations pertaining to biological functions of homeobox genes in legumes.

## Supporting Information

S1 Fig Phylogenetic tree showing clustering of *Arabidopsis thaliana* (AT), *Cicer arientinum* (Ca), *Glycine max* (Glyma), *Cajanus cajan* (*C*. *cajan*), *Medicago truncatula* (Medtr) and *Lotus japonicus* (LjSGA, LjT, chr) homeobox proteins based on full-length amino acid sequences. (PDF)

S2 Fig Phylogenetic tree showing clustering of *Arabidopsis thaliana* (AT), *Cicer arientinum* (Ca), *Glycine max* (Glyma), *Cajanus cajan* (*C*. *cajan*), *Medicago truncatula* (Medtr) and *Lotus japonicus* (LjSGA, LjT, chr) homeobox proteins based on homeobox domain sequences. (PDF)

S3 Fig Heat-map showing expression pattern of soybean homeobox genes in different tissues/organs/developmental stages. (TIF)

S4 Fig Heat-map showing expression patterns of homeobox genes in different tissues/organs/developmental stages of (A) *Lotus japonicus* (B) *Medicago truncatula*. (TIF)

S5 Fig Heat-map showing expression pattern of duplicate soybean homeobox genes in different tissues/organs/developmental stages. (TIF)

S6 Fig GOSlim term (biological process, molecular function and cellular component) assignment to chickpea genes harboring AH1 and/or AH2 motifs. (TIF)

S7 Fig The bar graphs depict the number of chickpea and soybean genes (harboring AH1 and/or AH2 motifs in their promoter regions) showing positive correlation (A) and negative correlation (B) with HD-Zip I and HD-Zip II genes of chickpea and soybean, on the basis of Pearson correlation coefficient score. (TIF)

S1 Table List of homeobox genes identified from chickpea, soybean, *Medicago*, *Lotus* and pigeonpea. (XLS)

S2 Table List of primer sequences of chickpea homeobox genes used in qRT-PCR analysis. (DOC)

S3 Table *Cis*-regulatory elements present in the promoter sequences of homeobox genes in chickpea and soybean. (XLS)

S4 Table Ratio of Ka/Ks and distribution of duplicated soybean homeobox genes in respective blocks as obtained from Plant Genome Duplication Database (PGDD). (XLS)

S5 Table List of coexpressed genes [harboring AH1 and/or AH2 motif(s)] with HD-Zip I and HD-Zip II homeobox genes in chickpea (A) and soybean (B). The coexpressed genes were identified on the basis of Pearson correlation coefficient score (≥ 0.7).
(XLSX)
