# Supplementary material for: Genome-Wide Analysis of Homeobox Gene Family in Legumes: Identification, Gene Duplication and Expression Profiling
Source: PLoS One. 2015 Mar 6;10(3):e0119198. doi: 10.1371/journal.pone.0119198 (PMC4352023; doi:10.1371/journal.pone.0119198)

**Figure S1.** Phylogenetic tree showing clustering of *Arabidopsis thaliana* (AT), *Cicer arientinum* (Ca), *Glycine max* (Glyma), *Cajanus cajan* (C. cajan), *Medicago truncatula* (Medtr) and *Lotus japonicus* (LjSGA, LjT, chr) homeobox proteins. The unrooted phylogenetic tree was obtained after multiple sequence alignment. Bootstrap support was based on 1,000 replicates and values have been indicated at appropriate branches. Different classes of homeobox gene family constituting separate clades have been shaded with different colors. Sequence IDs are given on each branch.

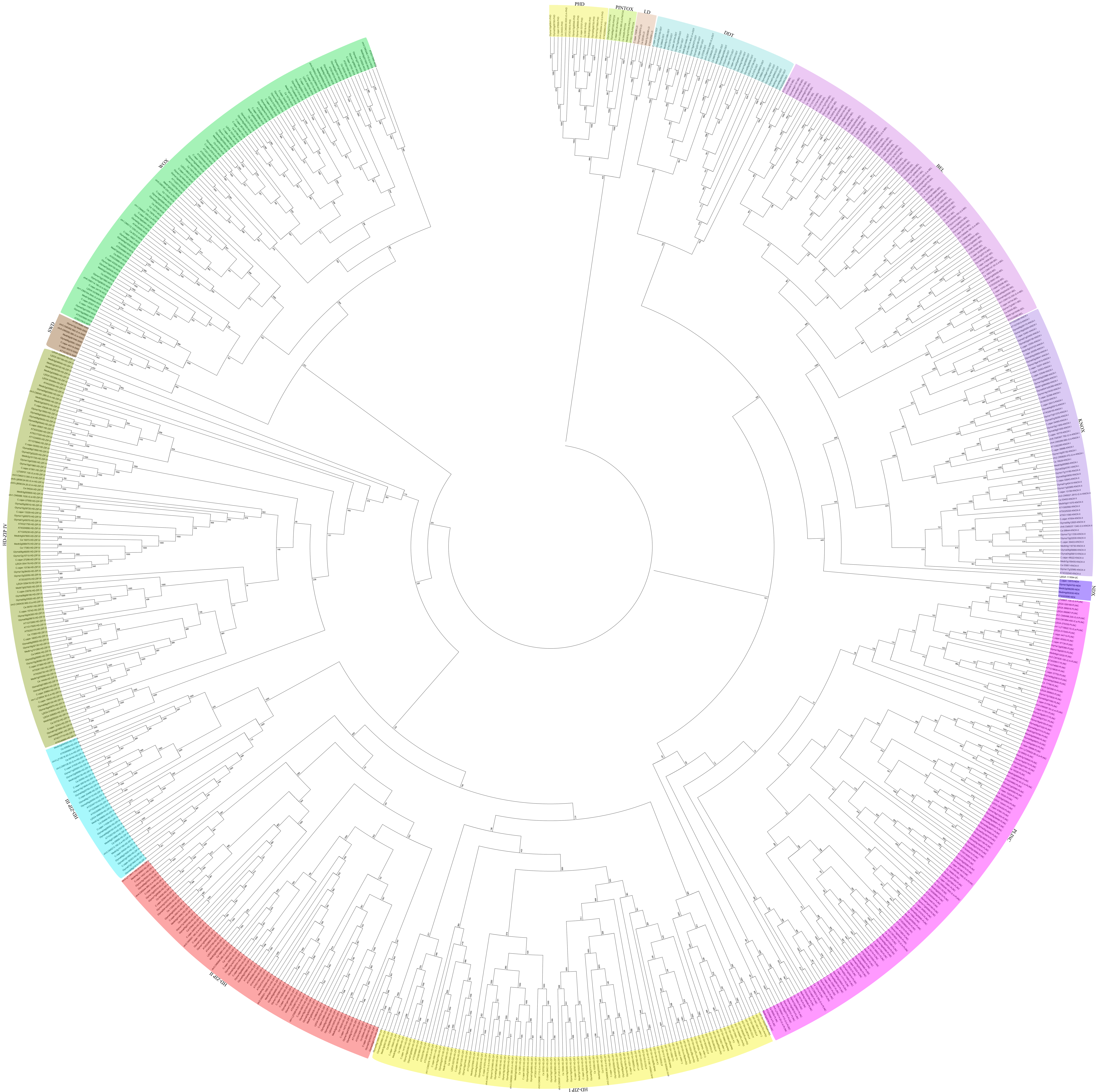

Supplement: S1 Fig — (PDF) [file pone.0119198.s001.pdf]
