# Supplementary figures and images for: Genome-Wide Analysis of Homeobox Gene Family in Legumes: Identification, Gene Duplication and Expression Profiling
Source: PLoS One. 2015 Mar 6;10(3):e0119198. doi: 10.1371/journal.pone.0119198 (PMC4352023; doi:10.1371/journal.pone.0119198)

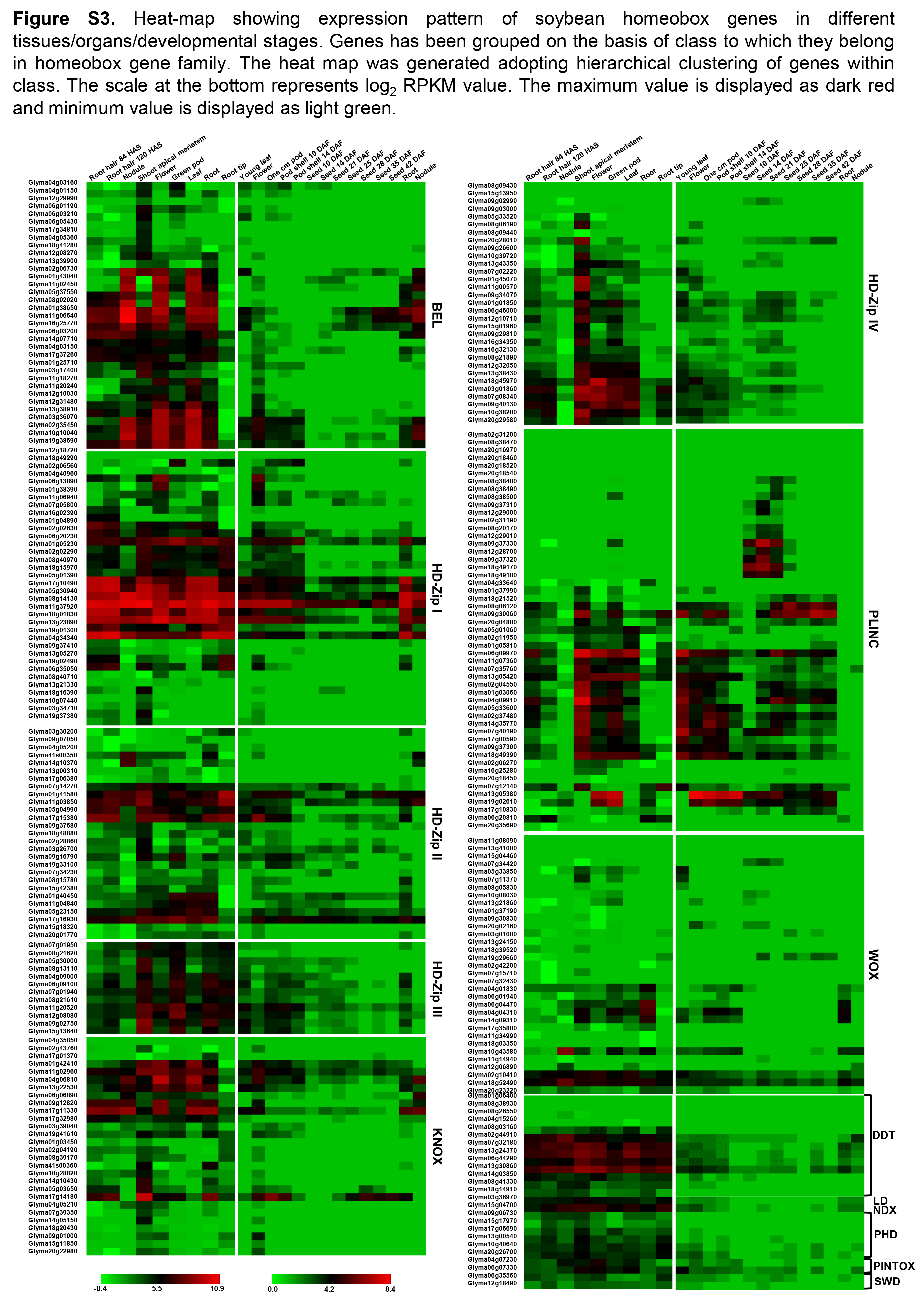

Supplement: S3 Fig — (TIF) [file pone.0119198.s003.tif]

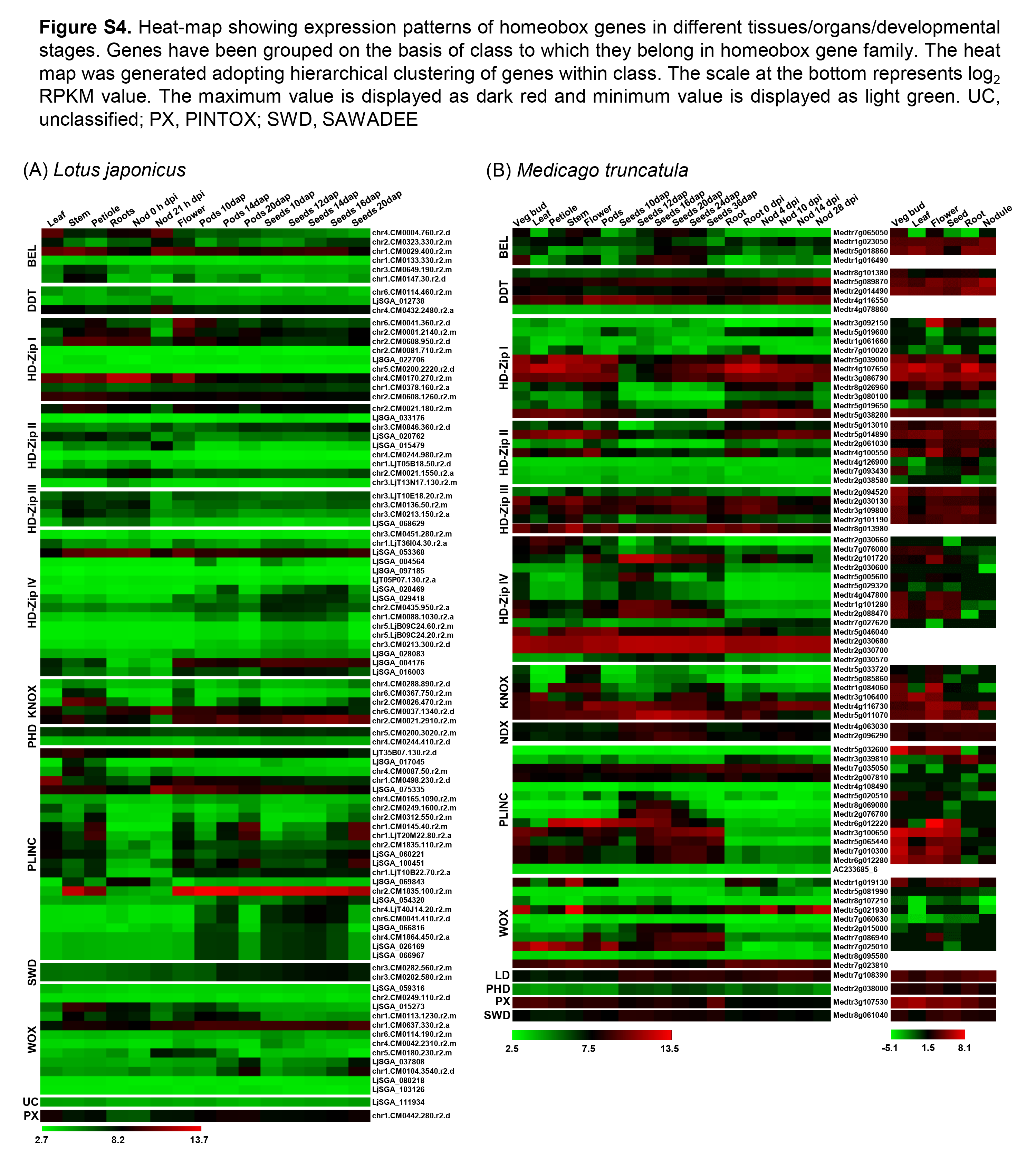

Supplement: S4 Fig — (TIF) [file pone.0119198.s004.tif]

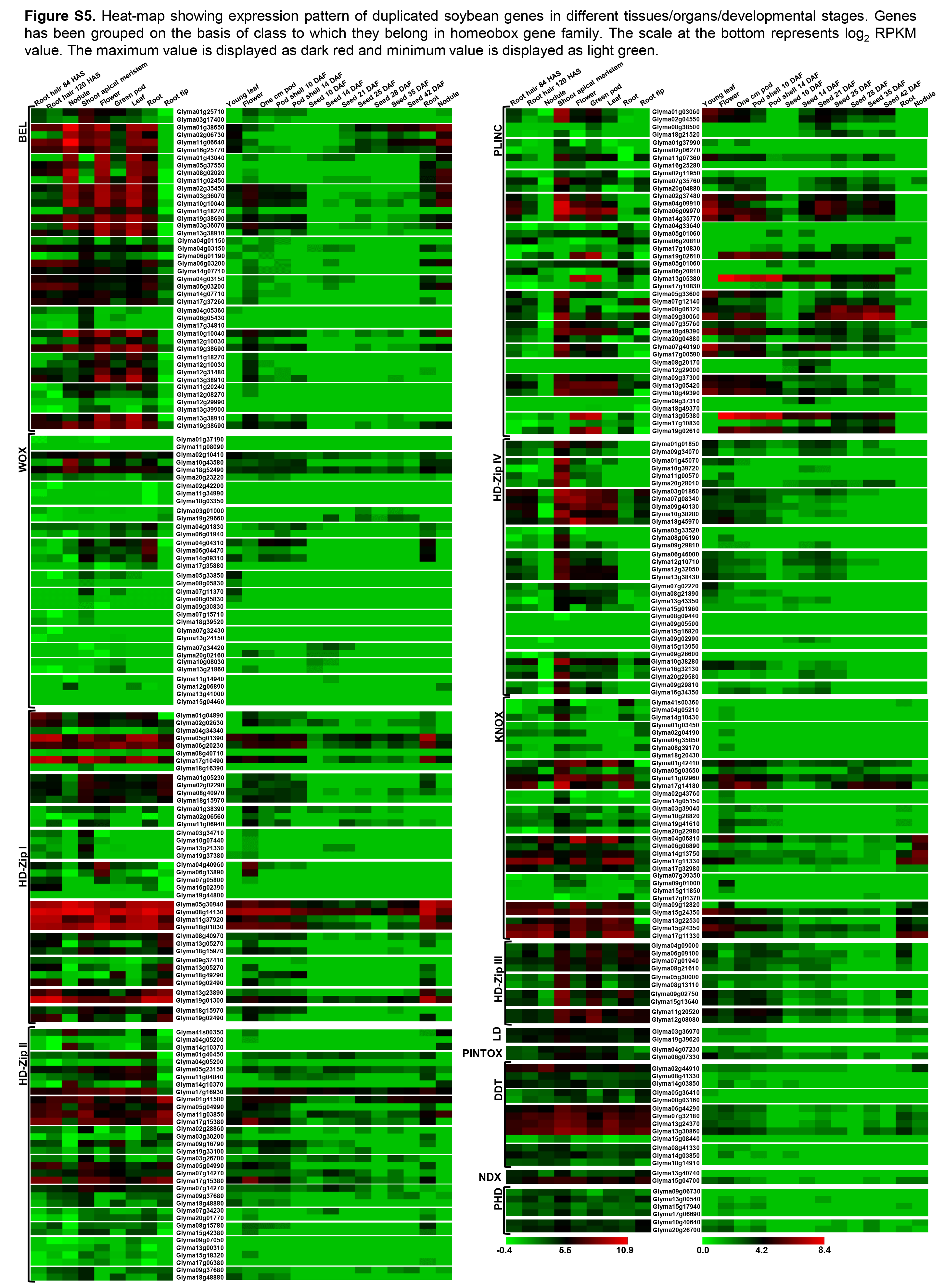

Supplement: S5 Fig — (TIF) [file pone.0119198.s005.tif]

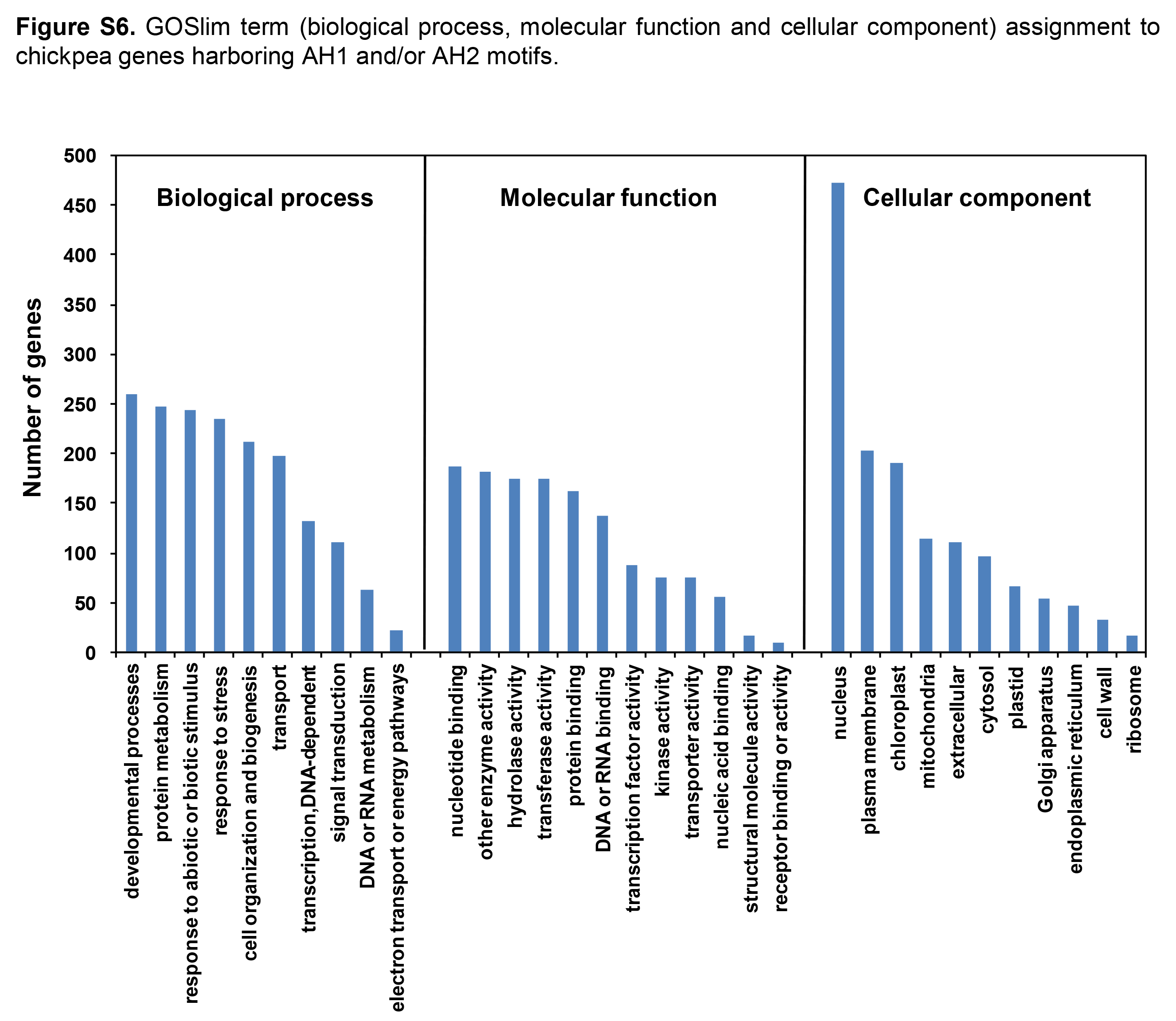

Supplement: S6 Fig — (TIF) [file pone.0119198.s006.tif]

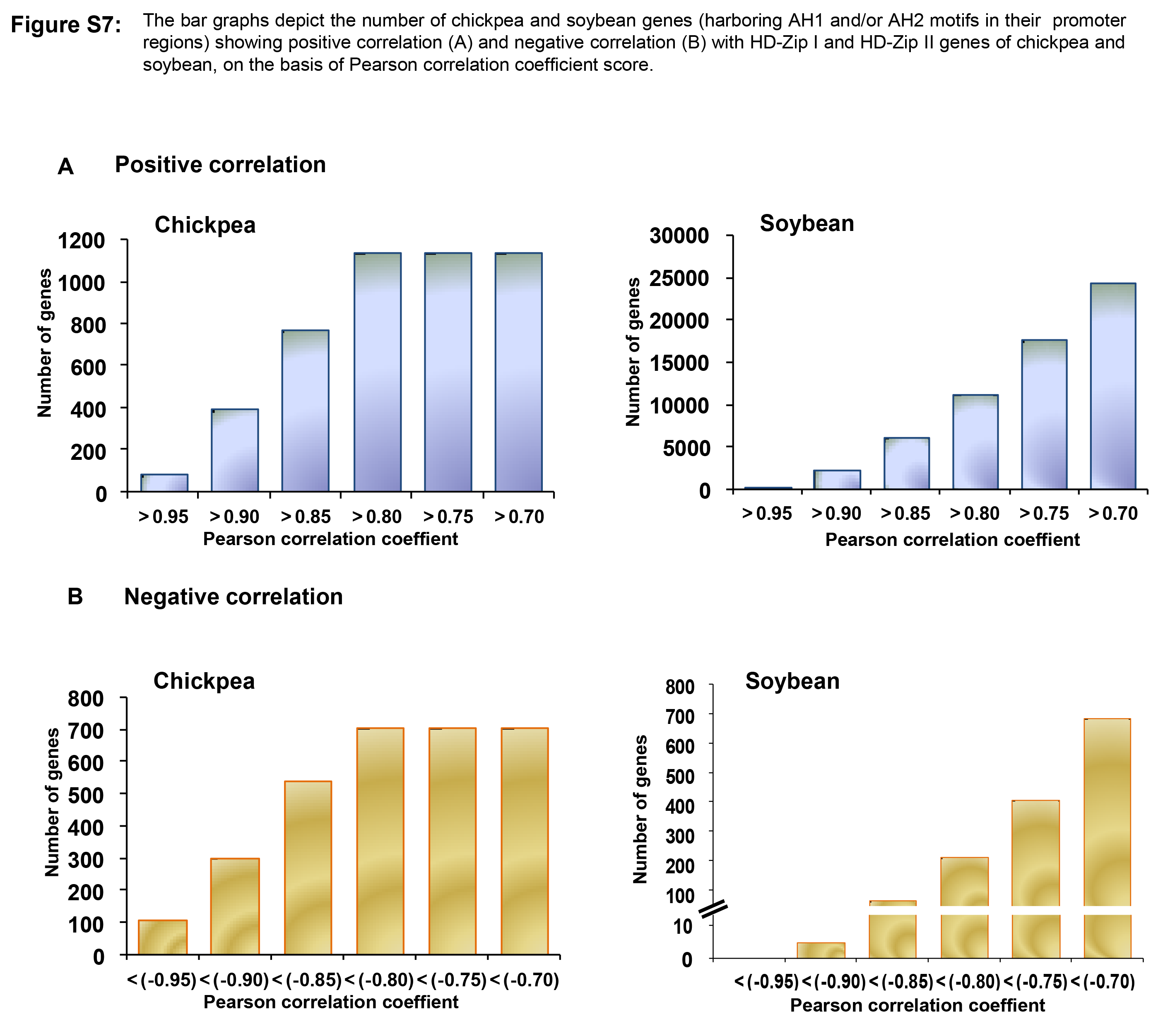

Supplement: S7 Fig — (TIF) [file pone.0119198.s007.tif]
