# Supplementary material for: Genome-Wide Analysis of Homeobox Gene Family in Legumes: Identification, Gene Duplication and Expression Profiling
Source: PLoS One. 2015 Mar 6;10(3):e0119198. doi: 10.1371/journal.pone.0119198 (PMC4352023; doi:10.1371/journal.pone.0119198)
Supplement: S2 Table — (DOC) [file pone.0119198.s009.doc]

**Table S2.** List of primer sequences of chickpea homeobox genes used in quantitative RT-PCR analysis.

| **Gene identifier** | **Forward Sequence** | **Reverse Sequence** |
| --- | --- | --- |
| Ca_00550 | CAACTGGTCTTTTGTTTGATGGAT | CAAAACACATGAGACAAAGCTGAA |
| Ca_01318 | GAGAACATGTCAGGCTTTTGCA | CACTTTGTGCCACTGCTACCA |
| Ca_03507 | TGAAAGAGTCGACCCAAGTAATAGC | GTGGGTCCACCACTTCCATT |
| Ca_04025 | TGTTGTTCATTGTTGGATTTTAAGTG | CCCACACACGACAACATTTAGG |
| Ca_04604 | TCTTCCAACTGCAAAACTCACAA | TTGATCTTCTGAATGGTGCATGA |
| Ca_06148 | CAAGCACACAGTGGAAGATCTCA | TGGAAAGCAAAGAAGCCATAGTG |
| Ca_06937 | TGCTGCAGATGTTACTGTTCCA | TGCCTCCTCACCAACACCTT |
| Ca_11206 | CAAAGCCCCATATTCACTTCTACA | GCCCTAGCTAGCAATAGTTGCAA |
| Ca_17288 | AATGAAATGGGTGGCAATGAA | TTGAGCAGCAAACCTCTTTCTG |
| Ca_17383 | CGAGGAGGTGTAGGGAACTTAGG | TCGAGAAGGAATGTTGCAAGGT |
| Ca_18320 | AAGAAGCGGCATAGAACGAAGT | TTTCTCAGCCAATTCCAACATCT |
